# Risk Factors Associated With Post-stroke Epilepsy: A Narrative Review

**DOI:** 10.7759/cureus.92134

**Published:** 2025-09-12

**Authors:** Nethra Somannagari, Alousious Kasagga, Aayam Sapkota, Gichin Changaramkumarath, Jane M Abucha, Mekdes M Wollel, Paolo S Chavez Cavalie

**Affiliations:** 1 Neurology, Gandhi Medical College, Hyderabad, IND; 2 Pathology, Peking University, Beijing, CHN; 3 Pathology, California School of Podiatric Medicine, Oakland, USA; 4 Internal Medicine, Chongqing Medical University, Chongqing, CHN; 5 Internal Medicine, American University of Integrated Sciences, Phoenix, USA; 6 Pediatrics and Child Health, University of Gondar, Gondar, ETH; 7 Surgery, Universidad Peruana de Ciencias Aplicadas, Lima, PER

**Keywords:** epilepsy, post-stroke complications, post-stroke epilepsy, risk factors‎, stroke

## Abstract

Post-stroke epilepsy (PSE) is a condition that impacts a notable portion of stroke survivors and is linked to worse health outcomes, such as increased risk of death and longer disability. PSE refers to seizures that occur unexpectedly after the first week following a stroke and result from several biological processes, including inflammation and damage from overactive nerve signaling. Existing prediction tools help identify high-risk patients but can be limited by inconsistent evaluation of risk factors. Twenty-seven studies were selected from databases including PubMed, Cochrane Library, and EBSCO using Medical Subject Headings (MeSH) terms and keywords related to stroke, epilepsy, and risk factors. Studies published between 2020 and 2025 were included based on predefined eligibility criteria. During the analysis, a wide range of risk factors were identified, including patient demographics (age, sex, comorbidities, genetic predisposition), stroke features (cortical involvement, lesion size, acute symptomatic seizures), diagnostic findings (early electroencephalogram abnormalities, structural changes on magnetic resonance imaging), and biochemical markers (blood proteins, microRNAs, TNFSF-14). Early interventions, such as hematoma evacuation and thrombectomy, also show potential in reducing PSE risk, while emerging biomarkers, including specific blood proteins and miRNAs, offer promise for early diagnosis. It is recognized that both the occurrence and the timing of early seizure activity may be important for patient outcomes. Interestingly, deep grey matter loss and asymmetrical perivascular spaces were also associated with a higher risk of PSE. Despite these advances, considerable variability remains a challenge across studies. Together, these insights highlight the need for a unified approach to develop better tools for assessing PSE risk, with the ultimate goal of preventing this complication during stroke care.

## Introduction and background

Stroke is a significant cause of morbidity and mortality worldwide, and its complications, both immediate and long-term, play an essential role in poor outcomes. These range from acute events like cerebral edema, deep vein thrombosis, and pneumonia to chronic issues such as post-stroke epilepsy (PSE), motor deficits, and depression, of which PSE is seen in 2-20%of stroke patients [1,2]. PSE contributes to high mortality, a significant decline in functional outcome, long-term disability, and dementia risk [3-5]. Given its considerable burden on patients, PSE is a critical complication of stroke that warrants greater clinical attention. 

PSE refers to the occurrence of unprovoked epileptic seizures that begin more than seven days after a stroke event. According to the International League Against Epilepsy (ILAE), one such late-onset seizure (with a high risk of recurrence) qualifies as epilepsy in this context [6]. Many pathophysiological mechanisms contribute to the development of epilepsy in stroke patients, such as the neuroinflammation triggered by stroke, glutamate excitotoxicity released by the ischemic brain cells, reactive gliosis forming epileptogenic foci, and ion channel dysfunctions [7]. It has a wide range of clinical manifestations, such as generalized tonic-clonic, focal aware, or secondary generalized [8]. 

Previous studies have introduced scoring models, like SeLECT and its variants and CAVE-S in ischemic and hemorrhagic stroke, respectively [2]. SeLECT stands for Severity of stroke, Large-artery atherosclerotic etiology, Early seizures, Cortical involvement, and Territory of the middle cerebral artery, while CAVE-S includes Cortical involvement, Age <65 years, Volume of hemorrhage >10 mL, Early seizures, and Severity of stroke [9-11]. These tools effectively identify patients at high risk of developing PSE by assigning points to the key factors described above. These scoring systems offer helpful guidance, yet the prediction of PSE requires a more comprehensive clinical evaluation beyond SeLECT and CAVE-S scores alone. A notable gap in the literature is the lack of consistency in the risk factors evaluated across studies, which results in fragmented evidence. However, the potential for future predictive models to bridge this gap and improve stroke care is promising. This inconsistency can be overcome by reviews that provide a more comprehensive perspective on PSE risk factors. 

PSE is preventable in some cases, but predicting who will develop it remains challenging. Given the significant burden of PSE on patients, caregivers, and healthcare systems, identifying and understanding its risk factors is paramount. Recognizing them at an early stage is critical for mitigating the long-term impact of PSE and ensuring prompt management through targeted surveillance. 

A broad spectrum of risk factors, including patient-specific variables, stroke-related characteristics, neuroimaging markers, and therapeutic interventions, are considered, making the risk profile more comprehensive and diverse. This review seeks to present a comprehensive overview of risk factors linked to PSE, assessing both currently used predictors and proposing novel factors for inclusion in future predictive models. A detailed risk framework needs to be established to support the use of existing models and to develop a clinically applicable model that can be seamlessly integrated into stroke care pathways.

## Review

Methods

This review was conducted using the SANRA (Scale for the Assessment of Narrative Review Articles) checklist to improve the quality [12]. The six key domains have been used to ensure that standards have been maintained during the writing process. The importance of this article has been justified, a concrete aim has been established, and a detailed description of the literature search was provided, using the key concepts, search terms and inclusion criteria. Many references were cited to support the statements made. Appropriate presentation of results and scientific reasoning was ensured [12].

Search Sources and Strategy

A comprehensive search was conducted across multiple electronic databases, including PubMed, Cochrane Library and EBSCO, to identify literature relevant to risk factors of PSE. A systematic search strategy was developed using keywords and Medical Subject Headings (MeSH terms) to identify relevant studies (Table 1). The research question focused on "Risk factors associated with the development of epilepsy in stroke survivors". 

**Table 1:** Search Strategy

Search Strategy	Database Used	Number of Papers Identified
((( "Stroke/complications"[Mesh] OR "Stroke/history"[Mesh] )) AND ( "Epilepsy/diagnosis"[Mesh] OR "Epilepsy/etiology"[Mesh] )) AND "Risk Factors"[Mesh]	PubMed(MeSH)	34
risk factors and stroke and epilepsy	PubMed	283
((((stroke[Title/Abstract]) AND (epilepsy[Title/Abstract])) AND (risk factors[Text Word])) AND (english[Language])) AND (("2020/01/01"[Date - Publication] : "3000"[Date - Publication])))	PubMed	196
risk factors AND stroke AND epilepsy	Cochrane Library	12
risk factors AND stroke AND epilepsy	EBSCO	32

Key Concepts and Search Terms

The key concepts of "stroke," "epilepsy," and "risk factors" were searched using the MeSH terms "Stroke/complications" OR "Stroke/history," "Epilepsy/diagnosis" OR "Epilepsy/etiology," and "Risk Factors." These were combined with "AND" to retrieve articles addressing all three topics (Table 1).

Search Queries

The PubMed Medical Subject Headings (MeSH) search query applied was (("Stroke/complications"[Mesh] OR "Stroke/history"[Mesh]) AND ("Epilepsy/diagnosis"[Mesh] OR "Epilepsy/etiology"[Mesh])) AND "Risk Factors"[Mesh]. In this query, the OR operator groups similar concepts within stroke and epilepsy categories, while the AND operator connects stroke, epilepsy, and risk factor terms. The PubMed keyword search utilised the following terms: risk factors AND stroke AND epilepsy. For searches limited to title, abstract, and publication date, the query was: ((((stroke[Title/Abstract]) AND (epilepsy[Title/Abstract])) AND (risk factors[Text Word])) AND (english[Language])) AND (("2020/01/01"[Date - Publication]: "3000"[Date - Publication])) (Table 1).

Study Screening

**Figure 1:**
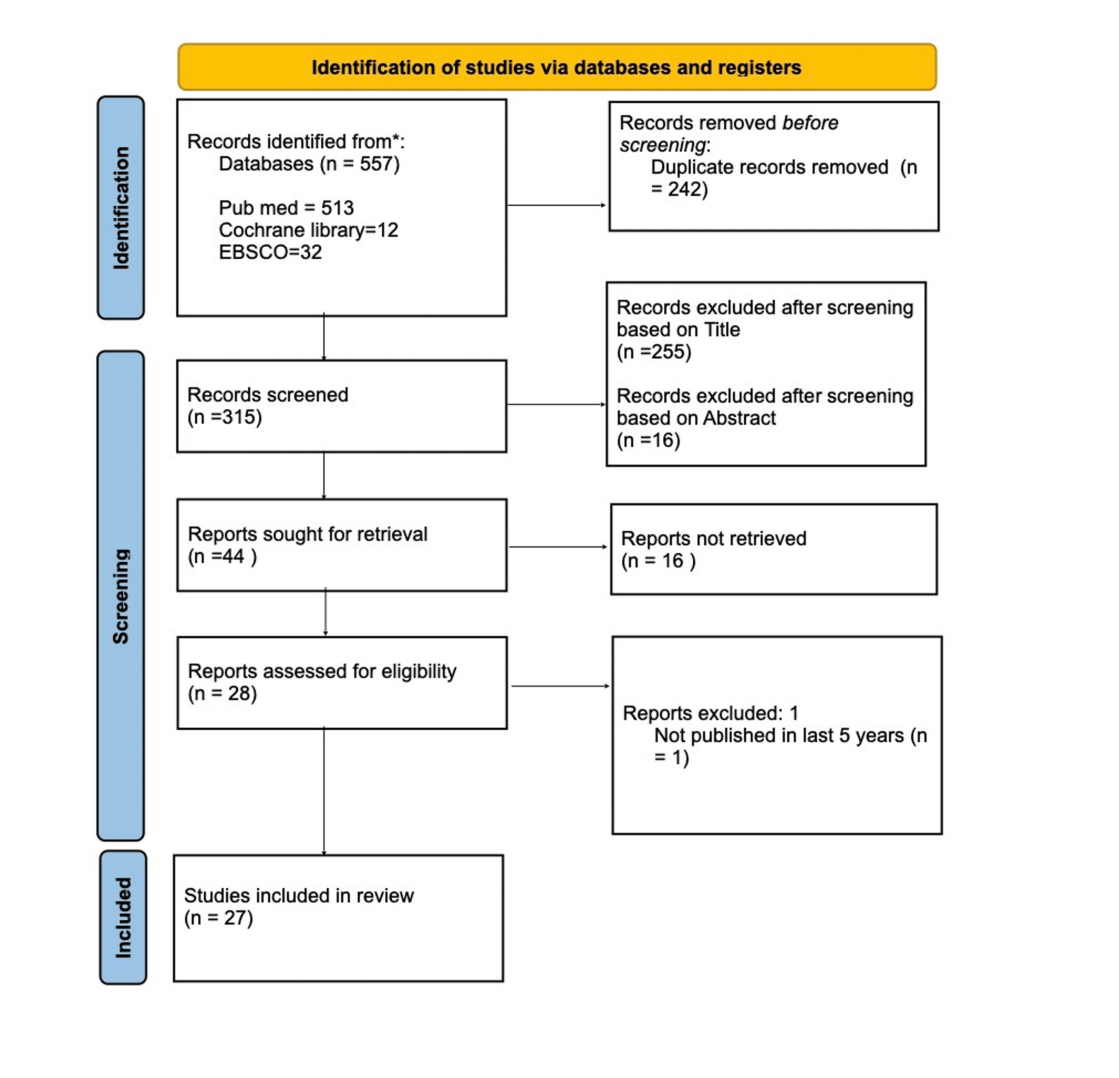
Flow Diagram of the Study Selection Process

Following the database searches, all identified records were imported into a reference management software. Duplicate records were removed, resulting in the exclusion of 242 duplicates. This left 315 unique records for initial screening. The screening process involved title and abstract screening along with full-text review. The titles and abstracts of the 315 unique records were screened for relevance to the research question. This stage led to the exclusion of 255 records based on their titles and an additional 16 documents based on their abstracts, resulting in a total of 271 exclusions. A total of 44 reports were sought for retrieval, and 16 of these reports could not be retrieved. The remaining 28 reports were assessed for eligibility. During the full-text assessment, one report was excluded because it was not published within the last five years. Ultimately, 27 studies were included in this narrative review (Figure 1).

Eligibility Criteria

Specific eligibility criteria were applied through the screening process to ensure the suitability and reliability of the included literature. Original research papers published in English between 2020 and 2025 were considered for inclusion. Human research across any age group was acknowledged. Studies were excluded if they were grey literature or if the patient population had pre-existing epilepsy before the stroke event.

Discussion

PSE is a significant complication of stroke and has a case fatality rate of approximately 27.4% [3]. Studies have found recurrence in seizures within the first year in nearly 23.7% of patients, which has led to a higher risk of decline in their functional outcome(11.8%) [5]. It has a profound impact on patients' quality of life; hence, the timely identification and management of the risk factors associated with it are necessary. Previous reviews of literature highlighted the need for a multidisciplinary paradigm in assessing PSE risk, integrating clinical, imaging, electrophysiological, and molecular data. Galovic et al. emphasize that clinical predictors, such as age, stroke severity, and cortical involvement, can be significantly enhanced by incorporating early electroencephalogram (EEG) findings and blood-based biomarkers into prognostic models, like SeLECT and CAVE [13]. Likewise, Wiśniewski and Jatužis highlight the multifactorial nature of late post-stroke seizures, not only clinical and neuroimaging markers, but also adjunctive data from EEG, genetic profiles, and serum biomarkers, to improve model accuracy is required [14]. A review of all the risk factors encompassing patient-specific characteristics, stroke features, diagnostic biomarkers and imaging findings is provided. This integrative approach is necessary for future risk scores to be effectively incorporated into clinical settings.

Patient-Specific and Genetic Risk Factors

Several studies across various patient populations describe certain patient-specific factors that increase the risk of PSE. A study with 13,471 patients of the age group 19-44 years in Taiwan found that behavioral risk factors like drug abuse (HR, 2.9; 95% CI, 1.53-5.50), smoking, alcohol use and certain underlying conditions like malignancy were associated with higher rates of PSE [15]. This underscores the need for comprehensive patient care, considering not only the stroke but also these lifestyle and comorbid factors. Younger patients with stroke had a higher risk for PSE (p = 0.001), with a median age of 39, which was also supported in a meta-analysis by Nandan et al., where children (age<18 years) were at a higher risk [15,16]. Patients with dyslipidemia showed a lower risk of PSE, which may be attributed to the use of statins in them, indicating their protective nature [15]. A case-control study by Waafi et al. in the Indonesian population found that men had a 3.35 times higher risk of PSE than women. However, when the EEG patterns were compared, women had a more abnormal pattern than men [17]. 

A Serbian study by Živadinović et al. in 267 patients noticed an increased risk of PSE in patients with underlying pulmonary and cardiovascular comorbidities like arrhythmias, pacemaker implantations, and a history of cardiac bypass surgery (p=0.02) [18]. Complications like uremia, a history of deep vein thrombosis, atrial fibrillation, hyperuricemia, cerebral hernia, hydrocephalus, coronary heart disease, diabetes, hyperlipidemia, and fatty liver were compared, of which hypertension was more strongly associated with the development of PSE [19]. A German cohort study identified a significant association between prolonged hospital stay and the subsequent development of PSE (HR = 1.02; 95% CI: 1.02-1.02; p <.001) [20]. This suggests that more extended hospitalisation may reflect greater complications, both of which could contribute to an increased PSE risk [20]. Specific genetic factors which increase the risk of epilepsy have also been associated with increased risk of PSE, which have been quantified by using a polygenic risk factor, indicating a genetic predisposition (OR, 1.13 {95% CI, 1.05-1.21}; P=0.002) [21]. The ALDH2 rs671 polymorphism and low serum magnesium levels, associated with the rs2274924 C allele polymorphism of the TRPM6 gene, may serve as a potential predictor of PSE by decreasing the seizure threshold [22]. This suggests a genetic contribution to PSE risk. Table 2 summarizes patient-specific and genetic risk factors.

**Table 2:** Summary of Risk Factors in Post-stroke Epilepsy PSE: post-stroke epilepsy; EEG; electroencephalogram; EPVS: enlarged periventricular space; HTN: hypertension; CHD: coronary heart disease; AF: atrial fibrillation; DM: diabetes mellitus; NSE: neuron-specific enolase; TNFSF-14: tumor necrosis factor superfamily 14; NIHSS: National Institutes of Health Stroke Scale; ALDH2: Aldehyde Dehydrogenase 2; TRPM6: Transient Receptor Potential Melastatin 6

Category	Risk Factor	Key Findings	Authors (year) [citation number]
Patient-Specific Factors	Young age (<44 years, children)	Increased risk of PSE among younger adults and children.	Do et al., 2022 [15]; Nandan et al., 2023 [16]
	Male sex	Higher PSE risk in men; EEG abnormalities are more common in women.	Waafi et al., 2023 [17]
	Lifestyle (smoking, alcohol, drugs)	Associated with increased risk of PSE.	Do et al., 2022 [15]
	Comorbidities (HTN, CHD, AF, DM, fatty liver)	Hypertension is the most significant comorbidity linked to PSE.	Živadinović et al., 2023 [18]; Liu et al., 2024 [19]
	Hospitalization duration	A more extended inpatient stay is a predictive factor.	Hardtstock et al., 2021 [20]
	Genetic predisposition	Polygenic risk scores, the TRPM6 and the ALDH2 rs671 polymorphism are linked to increased Susceptibility.	Clocchiatti-Tuozzo et al., 2024 [21]; Phan et al., 2022 [22]
Stroke Characteristics	Seizure timing (early vs. late)	Seizures within three days of stroke onset increase PSE risk.	Lin et al., 2021 [23]
	Hippocampal sclerosis	Structural changes like HS promote epileptogenesis.	Stancu et al., 2022 [24]
	Seizure type	Status epilepticus and focal-to-bilateral tonic-clonic seizures have the highest PSE risk.	Schubert et al., 2025 [25]
	Stroke subtype	Ischemic strokes are more frequently associated with PSE.	Dziadkowiak et al., 2021 [26]
	Hemisphere involvement	Left-sided strokes are more strongly associated with PSE.	Schubert et al., 2025 [25]; Winder et al., 2023 [27]
	Stroke severity and recurrence	NIHSS scores greater than five are associated with an increased risk of PSE.	Do et al., 2022 [15]
	Stroke etiology	Undetermined etiologies were associated with a higher risk of PSE.	Liu et al., 2024 [17]
Diagnostic Markers	Lesion characteristics	Larger lesion volume and cortical involvement, especially in the temporal and occipital lobes.	Winder et al., 2023 [27]
	Perivascular space asymmetry	EPVS asymmetry in the centrum semiovale is linked with PSE.	Yu et al., 2022 [28]
	Deep grey matter volume loss	Thalamus and basal ganglia atrophy are linked with pediatric PSE.	Vaher et al., 2023 [29]
	EEG abnormalities	Focal slowing and epileptiform discharges are predictive of PSE.	Tatilo et al., 2024 [30]
Interventions	Hematoma evacuation	>70% reduction in PSE risk when a hematoma volume ≥10 mL is surgically removed.	Welte et al., 2023 [31]
	Thrombectomy / Revascularisation	Reduced PSE risk in severe stroke cases following early intervention.	Ebbesen et al., 2024 [32]
Biomarkers	Inflammatory markers(S100B,NSE, TNFSF-14)	Elevated levels post-stroke correlate with PSE onset.	Liang et al., 2021 [33]; Wen et al., 2022 [34]; Vasilieva et al., 2023 [35]; Abraira et al., 2024 [36]
	Genetic/epigenetic markers (miR-485-5p)	Lower miR-485-5p expression is associated with higher PSE risk.	Chai et al., 2024 [37]

Stroke Characteristics and Early Seizure Activity

Acute symptomatic seizures, seizures within seven days of a stroke, have also been noted to have a strong correlation with PSE OR=2.00 (1.28-3.145) [38]. However, evidence also suggests that seizures after the first three days of stroke have a greater risk of developing PSE, indicating that the timing of seizures also plays a crucial role in PSE (HR 3.84, 95% CI 1.77-8.31, p = 0.001) [23]. A retrospective study by Stancu et al. shows that in these acute symptomatic seizure patients, hippocampal sclerosis contributes to the development of PSE (p < 0.00086) [24]. When these seizure types were compared, status epilepticus (aHR, 9.6 {95% CI, 3. 5-26. 7}; P<0. 001) in the first seven days of stroke was associated with higher risks of PSE followed by focal to bilateral tonic-clonic seizure (aHR, 3.4 {95% CI, 1. 9-6. 3}; P<0. 001) [25]. However, a study by Dziadkowiak et al. found that generalized tonic-clonic seizures were most commonly observed in patients who had experienced severe strokes, as indicated by low ASPECTS scores (0-4), reflecting extensive early ischemic damage (p = 0.01430) [26]. The involvement of the left hemisphere of the brain more than the right hemisphere in PSE has also been mentioned in the literature [25,27]. In a retrospective analysis of 164 patients, the majority of PSE cases (n=144) developed following ischemic stroke, while a smaller proportion (n=20) occurred after hemorrhagic stroke. This distribution suggests the higher prevalence of PSE among ischemic stroke survivors, highlighting stroke subtype as a potential risk factor [26]. Stroke severity and recurrence were both identified as significant risk factors for PSE [15]. When the National Institutes of Health Stroke Scale (NIHSS) scores were compared, scores of five to nine were associated with a 1.4-fold increase in risk, and scores greater than ten were associated with a 1.98-fold increase in risk of PSE [15]. Comparisons of stroke etiologies by Do et al. found no significant association between large or small vessel diseases and PSE in young adults [15]. In contrast, Liu et al. reported that well-defined etiologies, such as large artery atherosclerosis and cardioembolism, were associated with a lower risk of PSE. However, undetermined etiologies were associated with a higher risk due to their poor prognosis [19]. Table 2 summarizes the stroke characteristics and early seizure activity risk factors.

Diagnostic Indicators

Imaging studies like magnetic resonance imaging (MRI) and computed tomography (CT) were assessed among many stroke patients to identify potential neuroanatomical risk factors. Research by Winder et al. indicates that ischemic lesions in the cortex were seen in 90% of the PSE patients, mostly involving the left temporal and occipital association cortex [27]. Imaging also reveals that larger lesions have a higher risk of developing, indicating that the lesion volume is also a significant risk factor for PSE (p< 0.002) [28]. Evidence from MRI in acute ischemic stroke patients showed asymmetric enlarged perivascular spaces in the centrum semiovale as a novel radiological marker for PSE, which was quantified by EPVS (enlarged periventricular spaces) scores (OR = 3.709, CI {1.508-9.123}, p = 0.004) [28]. These asymmetric changes suggest a localized, hemisphere-specific environment of accumulating inflammatory mediators that can lead to neuronal hyperexcitability. In a pediatric cohort study, MRI analysis revealed that structural volume loss in the thalamus and basal ganglia was present in post-perinatal stroke epilepsy, which indicates the role of deep grey matter structures in maintaining cortical stability [29]. This could serve as a useful risk factor for early screening and identification of post-perinatal stroke epilepsy in children. Early EEG abnormalities, like focal slowing and epileptiform discharge, were predicted by using continuous EEG in 81 high-risk ischemic stroke patients predicted to have epileptogenesis (odds ratio 18.8; p = 0.002) [30]. These findings suggest that early post-stroke EEG changes may serve as valuable indicators for identifying patients at increased risk of developing PSE. The diagnostic indicator risk factors are summarized in Table 2. 

Interventional and Biomolecular Contributions to PSE Risk

In a study with 587 ICH (intracranial hemorrhage) patients, 23.7% of patients developed epilepsy [31]. The ICH volume of greater than 10ml was a noted risk factor in developing epilepsy, and the surgical evacuation of these hematomas was associated with almost a 70% decrease in epilepsy risk [31]. Hence, a timely surgical evacuation may play a protective role by reducing the mass effect. A study in Denver identified that revascularization, especially thrombectomy with thrombolysis, had a lower incidence of epilepsy in patients with severe stroke, suggesting that early restoration of cerebral perfusion can stop epileptogenesis (adjusted HR, 0.55 {95% CI, 0.41-0.73}) [32]. Interventional risk factors to PSE are summarized in Table 2.

A review was conducted on the inflammatory biomarkers, neurotransmitter biomarkers, genetic biomarkers, and neuroprotein biomarkers in PSE, identifying S100B, NSE (neuron-specific enolase) and miRNAs as being associated with PSE [33]. These findings are supported by a study conducted in China by Wen et al. with 156 patients, where S100B and NSE emerged as potential risk determinants for PSE [34]. A systematic review by Vasilieva et al. on miRNAs showed promising outcomes of their involvement in epilepsy, which can be attributed to the miRNAs' dysregulation after brain injuries like stroke [35]. The consistency of these biomarker findings across independent studies reinforces their potential utility as diagnostic or prognostic indicators in the early detection of PSE. In another group of PSE patients, blood biomarkers overexpressed in them were studied, of which TNFSF-14 (tumor necrosis factor superfamily 14) emerged as the most significant one (AUC 0.733, 95% CI 0.601-0.865, p=0.006). These findings support the underlying pathophysiological mechanism in which neuroinflammation contributes to seizure activity following a stroke, highlighting its role in the development of PSE [36]. A more specific study on miRNAs in PSE patients showed that miR-485-5p (OR = 6.61, 95% CI: 2.234-16.984, p < 0.0001) is capable of determining the risk for epilepsy in post-stroke patients and that lower levels of miR-485-5p have been seen in these patients [37]. A summary of biomolecular risk factors is provided in Table 2. 

Although there are advances in PSE risk stratification, some critical gaps remain, and a few areas remain unexplored. Several studies focus on the clinical risk factors, such as the stroke subtype or the cortical involvement. Still, more literature is needed on biomarkers, advanced imaging and interventions to develop a more integrated approach. Despite their utility in predicting PSE, the SeLECT and CAVE models have not yet been widely implemented in everyday clinical settings. Consequently, this limits our ability to fully characterise the spectrum of risk factors associated with PSE. While the body of literature on PSE continues to expand, studies dedicated explicitly to elucidating its risk factors remain limited. Targeted research in this area is needed for the early identification of high-risk individuals and to pave the way for effective primary prevention strategies. 

Certain limitations should be acknowledged in our study, as they may affect the interpretation of results. The most notable one is the heterogeneity across studies. Factors like the different follow-up duration for epilepsy after stroke, variability in the distribution of ischemic and hemorrhagic stroke patients across studies, geographic location and various age groups may impact the consistency of reported PSE incidence and risk factor correlations. Article searches are limited by language and usage of specific databases, which can result in the omission of data from other relevant articles published in languages other than English. It is important to note that the studies analysed may not have uniformly assessed all known or suspected risk factors for PSE. Possible publication bias and lack of involvement of grey literature can result in additional limitations. This selective focus in the literature could lead to an incomplete representation of the overall spectrum of risk factors associated with PSE.

## Conclusions

Addressing the risk factors associated with PSE is crucial for improving patient outcomes and optimizing resource allocation in post-stroke care. By considering the risk factors from existing models, we reviewed their usefulness in light of recent findings, which may support their ongoing clinical use in predicting PSE.

Newly identified risk factors not currently included in models indicate the need for a multifaceted approach. Incorporating biomarker assays, advanced imaging modalities such as MRI, and genetic profiling could increase the accuracy of future predictive models. Research should prioritize enhancing the practical application of these factors and informing risk-based management strategies for PSE.

## References

[1] Graham NS, Crichton S, Koutroumanidis M, Wolfe CD, Rudd AG (2013). Incidence and associations of poststroke epilepsy: the prospective South London Stroke Register. Stroke.

[2] Tanaka T, Ihara M, Fukuma K, Mishra NK, Koepp MJ, Guekht A, Ikeda A (2024). Pathophysiology, diagnosis, prognosis, and prevention of poststroke epilepsy: clinical and research implications. Neurology.

[3] Arntz RM, Rutten-Jacobs LC, Maaijwee NA, Schoonderwaldt HC, Dorresteijn LD, van Dijk EJ, de Leeuw FE (2015). Poststroke epilepsy is associated with a high mortality after a stroke at young age: follow-up of transient ischemic attack and stroke patients and unelucidated risk factor evaluation study. Stroke.

[4] Zelano J, Redfors P, Åsberg S, Kumlien E (2016). Association between poststroke epilepsy and death: a nationwide cohort study. Eur Stroke J.

[5] Yoshimura H, Tanaka T, Fukuma K (2022). Impact of seizure recurrence on 1-year functional outcome and mortality in patients with poststroke epilepsy. Neurology.

[6] Holtkamp M, Beghi E, Benninger F, Kälviäinen R, Rocamora R, Christensen H (2017). European Stroke Organisation guidelines for the management of post-stroke seizures and epilepsy. Eur Stroke J.

[7] Doria JW, Forgacs PB (2019). Incidence, implications, and management of seizures following ischemic and hemorrhagic stroke. Curr Neurol Neurosci Rep.

[8] Sarecka-Hujar B, Kopyta I (2019). Poststroke epilepsy: current perspectives on diagnosis and treatment. Neuropsychiatr Dis Treat.

[9] Ayaz MA, Ibrahim M, Sharma S, Zawar I (2024). To Select or not to Select: evaluating the effectiveness of the SeLECT Score in predicting the risk of post-stroke epilepsy: a systematic review (P6-1.002). Neurology.

[10] Meletti S, Cuccurullo C, Orlandi N (2024). Prediction of epilepsy after stroke: proposal of a modified SeLECT 2.0 score based on posttreatment stroke outcome. Epilepsia.

[11] Schubert KM, Schmick A, Stattmann M, Galovic M (2025). Prognostic models for seizures and epilepsy after stroke, tumors and traumatic brain injury. Clin Neurophysiol Pract.

[12] Baethge C, Goldbeck-Wood S, Mertens S (2019). SANRA-a scale for the quality assessment of narrative review articles. Res Integr Peer Rev.

[13] Galovic M, Ferreira-Atuesta C, Abraira L (2021). Seizures and epilepsy after stroke: epidemiology, biomarkers and management. Drugs Aging.

[14] Wiśniewski A, Jatužis D (2021). Multifactorial predictors of late epileptic seizures related to stroke: Evaluation of the current possibilities of stratification based on existing prognostic models—a comprehensive review. Int J Environ Res Public Health.

[15] Do PT, Chen LY, Chan L, Hu CJ, Chien LN (2022). Risk factors for postischemic stroke epilepsy in young adults: a nationwide population-based study in Taiwan. Front Neurol.

[16] Nandan A, Zhou YM, Demoe L, Waheed A, Jain P, Widjaja E (2023). Incidence and risk factors of post-stroke seizures and epilepsy: systematic review and meta-analysis. J Int Med Res.

[17] Waafi AK, Husna M, Damayanti R, Setijowati N (2023). Clinical risk factors related to post-stroke epilepsy patients in Indonesia: a hospital-based study. Egypt J Neurol Psychiatr Neurosurg.

[18] Živadinović B, Lučić Prokin A, Živadinović J, Stojanov A (2023). Risk factors for the development of symptomatic epilepsy in patients diagnosed with stroke. Acta Med.

[19] Liu J, He H, Wang Y (2024). Predictive models for secondary epilepsy in patients with acute ischemic stroke within one year. Elife.

[20] Hardtstock F, Foskett N, Gille P (2021). Poststroke epilepsy incidence, risk factors and treatment: German claims analysis. Acta Neurol Scand.

[21] Clocchiatti-Tuozzo S, Rivier CA, Misra S (2024). Polygenic risk of epilepsy and poststroke epilepsy. Stroke.

[22] Phan J, Ramos M, Soares T, Parmar MS (2022). Poststroke seizure and epilepsy: a review of incidence, risk factors, diagnosis, pathophysiology, and pharmacological therapies. Oxid Med Cell Longev.

[23] Lin R, Yu Y, Wang Y (2021). Risk of post-stroke epilepsy following stroke-associated acute symptomatic seizures. Front Aging Neurosci.

[24] Stancu P, De Stefano P, Vargas M, Menetre E, Carrera E, Kleinschmidt A, Seeck M (2022). Acute symptomatic seizures and hippocampal sclerosis: the major contributor for post-stroke epilepsy?. J Neurol.

[25] Schubert KM, Zieglgänsberger D, Bicciato G (2025). Association of the timing and type of acute symptomatic seizures with poststroke epilepsy and mortality. Stroke.

[26] Dziadkowiak E, Guziński M, Chojdak-Łukasiewicz J, Wieczorek M, Paradowski B (2021). Predictive factors in post-stroke epilepsy: Retrospective analysis. Adv Clin Exp Med.

[27] Winder K, Bobinger T, Seifert F (2023). Incidence, temporal profile and neuroanatomic correlates of poststroke epilepsy. J Neuroimaging.

[28] Yu N, Sinclair B, Posada LM (2022). Asymmetric distribution of enlarged perivascular spaces in centrum semiovale may be associated with epilepsy after acute ischemic stroke. CNS Neurosci Ther.

[29] Vaher U, Ilves N, Ilves N (2023). The thalamus and basal ganglia are smaller in children with epilepsy after perinatal stroke. Front Neurol.

[30] Tatillo C, Legros B, Depondt C (2024). Prognostic value of early electrographic biomarkers of epileptogenesis in high-risk ischaemic stroke patients. Eur J Neurol.

[31] Welte TM, Steidl J, Stritzelberger J (2023). Surgical hematoma evacuation of cortical intracerebral hemorrhage ≥10 ml reduces risk of subsequent epilepsy by more than 70%: a retrospective monocenter study. Eur J Neurol.

[32] Ebbesen MQ, Dreier JW, Lolk K, Andersen G, Johnsen SP, Zelano J, Christensen J (2024). Revascularization therapies for ischemic stroke and association with risk of epilepsy: a Danish nationwide register‐based study. J Am Heart Assoc.

[33] Liang M, Zhang L, Geng Z (2021). Advances in the development of biomarkers for poststroke epilepsy. Biomed Res Int.

[34] WenF WenF, Lizhi Yu, Chengcai Xia, Zhenghua Gu (2022). Development and study of S100 calcium-binding protein B and Neuron-specific enolase-based predictive model for epilepsy secondary to cerebral infarction. Cell Mol Biol (Noisy-le-grand).

[35] Vasilieva AA, Timechko EE, Lysova KD, Paramonova AI, Yakimov AM, Kantimirova EA, Dmitrenko DV (2023). MicroRNAs as potential biomarkers of post-traumatic epileptogenesis: a systematic review. Int J Mol Sci.

[36] Abraira L, López-Maza S, Quintana M (2024). Exploratory study of blood biomarkers in patients with post-stroke epilepsy. Eur Stroke J.

[37] Chai Q, Chen X, Min L, Li H, Sun Y, Bu C (2024). Serum miR-485-5p expression and clinical significance in epilepsy secondary to cerebral infarction. Folia Neuropathol.

[38] Alemany M, Nuñez A, Falip M (2021). Acute symptomatic seizures and epilepsy after mechanical thrombectomy. A prospective long-term follow-up study. Seizure.

